# Evaluating the tailored implementation of a multisite care navigation service for mental health in rural and remote Australia (The Bridging Study): protocol for a community-engaged hybrid effectiveness-implementation study

**DOI:** 10.1186/s13012-024-01391-7

**Published:** 2024-09-04

**Authors:** Zephanie Tyack, Steven McPhail, Gregory A. Aarons, Kelly McGrath, Andrew Barron, Hannah Carter, Sarah Larkins, Adrian Barnett, Eloise Hummell, Ruth Tulleners, Olivia Fisher, Gillian Harvey, Lee Jones, Kate Murray, Bridget Abell

**Affiliations:** 1 Australian Centre for Health Services Innovation (AusHSI) and Centre for Healthcare Transformation, School of Public Health and Social Work, Queensland University of Technology, Brisbane, QLD Australia; 2 Clinical Informatics Directorate, Metro South Health, Brisbane, QLD Australia; 3 Department of Psychiatry, University of California San Diego, La Jolla, CA USA; 4 University of California San Diego Altman Clinical and Translational Research Institute Dissemination and Implementation Science Center, La Jolla, USA; 5 Health Services Research, Wesley Research Institute, Brisbane, QLD Australia; 6 College of Medicine and Dentistry, James Cook University, Townsville, QLD Australia; 7 Caring Futures Institute, College of Nursing and Health Sciences, Flinders University, Adelaide, Australia; 8 QIMR Berghofer Medical Research Institute, Statistics Unit, Brisbane, QLD Australia; 9 School of Psychology and Counselling, Queensland University of Technology, Brisbane, QLD Australia

**Keywords:** Implementation study, Hybrid effectiveness-implementation design, Mental health, EPIS framework, Community engagement, Participatory methods, Rural and remote, Adaptation, Scaling-up, Australia

## Abstract

**Background:**

A dramatic decline in mental health of people worldwide in the early COVID-19 pandemic years has not recovered. In rural and remote Australia, access to appropriate and timely mental health services has been identified as a major barrier to people seeking help for mental ill-health. From 2020 to 2021 a care navigation model, Navicare, was co-designed with rural and remote communities in the Greater Whitsunday Region of Central Queensland in Australia. The Exploration, Preparation, Implementation and Sustainment (EPIS) framework was used to design and guide multiple aspects of a multisite study, The Bridging Study, to evaluate the implementation of Navicare in Australia.

**Methods:**

A community-engaged hybrid effectiveness-implementation study design will focus on the tailored implementation of Navicare at three new sites as well as monitoring implementation at an existing site established since 2021. Study outcomes assessed will include sustained access as the co-primary outcome (measured using access to Navicare mental health referral services) and Proctor’s Implementation Outcomes of feasibility, acceptability, appropriateness, adoption, fidelity, implementation cost, and sustainability. Data collection for the implementation evaluation will include service usage data, community consultations, interviews, and workshops; analysed using mixed methods and guided by EPIS and other implementation frameworks. Pre-post effectiveness and cost-consequence study components are embedded in the implementation and sustainment phases, with comparison to pre-implementation data and value assessed for each EPIS phase using hospital, service, and resource allocation data. A scaling up strategy will be co-developed using a national roundtable forum in the final year of the study. Qualitative exploration of other aspects of the study (e.g., mechanisms of action and stakeholder engagement) will be conducted.

**Discussion:**

Our study will use tailoring to local sites and a community-engaged approach to drive implementation of a mental health care navigation service in rural and remote Australia, with expected benefits to mental healthcare access. This approach is consistent with policy recommendations nationally and internationally as building blocks for rural health including the World Health Organization Framework for Action on Strengthening Health Systems to Improve Health Outcomes.

**Trial registration:**

Prospectively registered on April 2, 2024, on the Australian New Zealand Clinical Trials Registry, no. ACTRN12624000382572. https://anzctr.org.au/Trial/Registration/TrialReview.aspx?id=386665&isReview=true.

Contributions to the literature

- The Bridging Study applies a community-engaged approach to all study activities including the selection of implementation strategies, with the potential to inform future research and drive sustainment.
- The study illustrates an approach that extends the use of EPIS to design, prepare for implementation, select implementation strategies, implement the intervention, adapt, evaluate implementation and inform scaling up. This could reduce the complexity of using multiple implementation frameworks.
- New ways of combining existing implementation frameworks have been proposed for an in-depth evaluation of adaptation as an implementation outcome, which may inform evaluation of this outcome in other studies.

## Background

The dramatic decline in mental health worldwide in the early COVID-19 pandemic years has not recovered, with the poorest mental health in younger people under 35 and in wealthier Anglosphere countries like Australia and the United Kingdom (UK) [1]. In Australia, mental ill-health and substance use disorders are leading contributors to total disease burden (15%), ranked second to the total burden from cancer (17%) [2]. One in five Australians experience mental illness each year [3]. There is notable inequity in this disease burden for those living outside major cities in rural and remote areas of Australia [4], who are disproportionately affected by severe mental health conditions and substance use disorders, including elevated rates of suicide [5]. A range of systemic and structural factors underpin this inequity including lack of local and equitable access to services [6–8].

The ability to access appropriate and timely mental health services is markedly more challenging in rural and remote areas than in urban centres [6] and was identified in initial co-design work as one of the greatest issues [9]. Reasons for this include strict eligibility criteria to access available services related to funding or age, insufficient funding [7] (for example, schemes that provide rebates or subsidies on a capped number of sessions such as a Medicare-rebated mental health care plan), and healthcare workforce challenges that manifest in restriction or suspension of services [10]. Solutions tailored to individuals and local contexts have been recommended [11] to account for the range of factors that underpin this mental healthcare inequity.

Care navigation models can address the issue of access to appropriate and timely care [12, 13] and have been implemented in mental health contexts including perinatal care [14] and for people with multiple chronic diseases [15]. These models can be tailored to individuals and sites and often consist of multiple components including screening, clinician support, and client support for connecting to social and mental health services. Despite implementation of care navigation models in routine care and some evidence of positive effects on mental health and quality of care outcomes, limited evaluation of the implementation effectiveness and scalability of these models of care has been conducted [14, 15].

From 2020 to 2021 a care navigation model, Navicare, was co-designed with local rural and remote communities in the Greater Whitsunday Region of Central Queensland in Australia, including with people with lived experience of mental ill-health, to address mental health care needs. The early phases of the Exploration, Preparation, Implementation, and Sustainment (EPIS) framework were used to guide the co-design process and EPIS’s dimensions (i.e., outer context, inner context, bridging factors, innovation factors, interconnections, linkages, and relationships) were used to identify barriers and facilitators (determinants) to implementation, core components of the intervention, and need for local tailoring as an implementation strategy prior to implementation [16]. Data collection involved 19 in-depth interviews with stakeholders and a community event with 30 participants.

Components of a potential intervention were presented and refined during the community event in a co-design process, considering the need to also adapt the intervention to local needs and context. As a result, four core components of the intervention (i.e., EPIS innovation factors) were identified as: (1) a local person who is a Care Navigator; (2) a local physical site where care coordination occurs and the Care Navigator is based; (3) online psychology and other services via supported telehealth; and (4) local champions to support adoption and sustainability. How these core components are implemented, however, can be tailored to the context of the local area. For example, a physical site may be a community centre or a pharmacy, or a new location may require fewer opening hours at the physical location and increased supported telehealth access. The role of the Care Navigator is to work at the frontline to assist community members to access mental health services, interacting with primary and community care services, private psychology services, hospitals and health services, and allied health services.

**Fig. 1:**
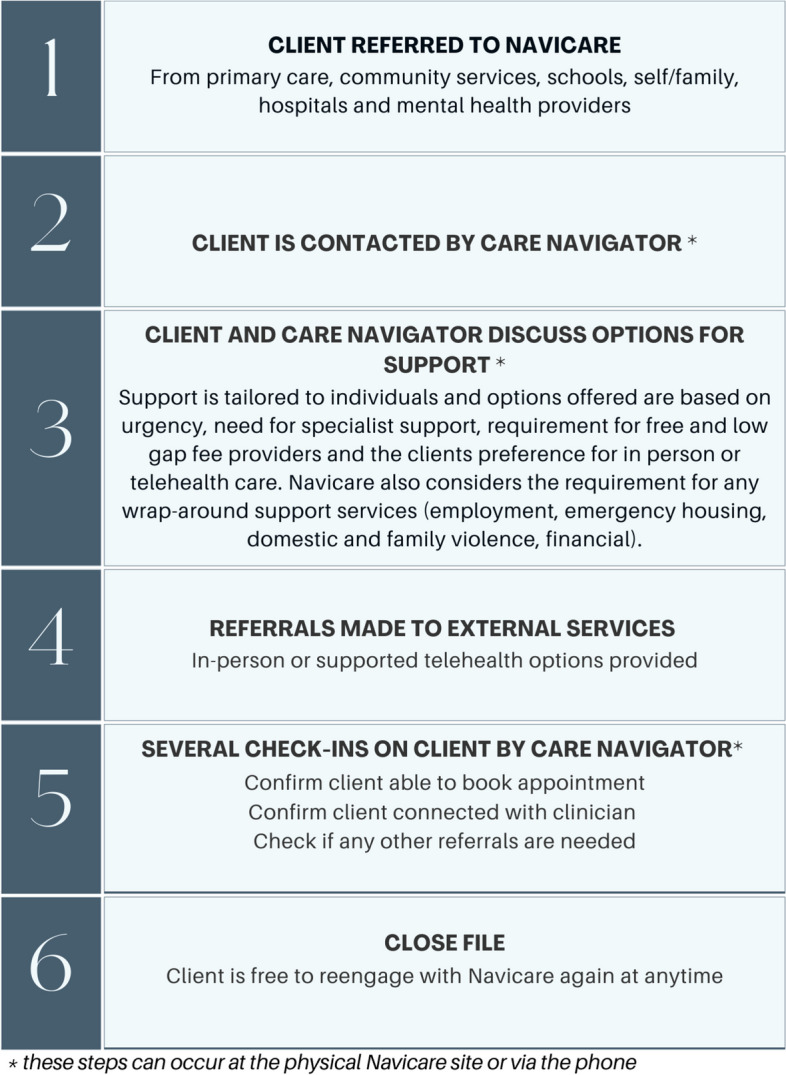
Navicare referral and care navigation processes

The feasibility of implementing Navicare has been demonstrated through establishment of a local physical site in Central Queensland, Care Navigators being based at that site, and over 600 service users accessing the service since 2021. There are no eligibility criteria for accessing Navicare beyond working or living in the catchment area. Community members can be referred or self-refer for reasons including anxiety, depression, self-harm, autism spectrum disorder, and alcohol and drug misuse. Navicare does not charge clients fees for care navigation services. Further details about the Navicare referral and care navigation processes can be found in Fig. 1.

EPIS has been used to design and guide many aspects of a multisite study, The Bridging Study, to evaluate the implementation of Navicare in Australia. Additional complementary frameworks have guided specific processes and reporting and are outlined in the methods. The overarching aim of this paper is thus to outline the application of EPIS and other implementation frameworks across the study that will: (1) evaluate access to Navicare; (2) determine the role of adaptation, community engagement, and contextualisation in the sustainability and scaling up of Navicare; (3) conduct a multisite, multilevel evaluation of the implementation and effectiveness of the Navicare service and potential for scaling up nationally across Australia; and (4) advance the meaningful use of implementation frameworks.

## Methods

### Study aims and hypothesis

#### Implementation component

- Aim 1: Determine whether implementation of the Navicare model sustains access (co-primary outcome) to mental health services in rural and remote settings in the post-implementation period compared to the implementation period.
- Aim 2: Determine the success (or otherwise) of implementing, scaling up and sustaining Navicare in four regional communities, and likely mechanisms of action.
- Aim 3: Understand the core and adaptable components of the Navicare model and implementation strategies across the study and within four communities in the Greater Whitsunday Region, Queensland.
- Aim 4: Provide conceptual evidence of the strengths and limitations of the EPIS framework when applied to rural and remote mental health in Australia and suggest refinement, complementary or alternate approaches for future studies.
- Hypothesis: The implementation of Navicare will sustain access (co-primary outcome) to mental health services in local rural and remote settings in the post-implementation period compared to the implementation period.

#### Effectiveness component

- Aim 5: Determine the effectiveness of Navicare in reducing time in emergency departments at a population level (co-primary outcome) and mental health related hospital admission outcomes (secondary outcomes).

#### Cost-consequences component

- Aim 6: Determine the cost consequences of the intervention and implementation strategies.

### Context

The study will implement and evaluate implementation of Navicare at an existing site and three new sites in the Greater Whitsunday Region in rural and remote Queensland. The Region has a population of 186,512 people and covers 90,354 square kilometres across several local government areas [17]. New sites that are the focus of the effectiveness component of the study will be determined as part of the study process based on community consultations, along with assessment of contextual factors and site readiness.

### Study design

**Fig. 2:**
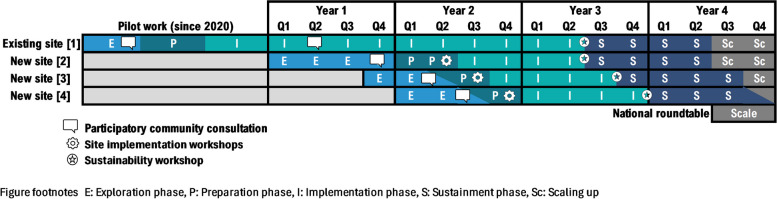
Timeline for each EPIS phase at each site

A pre-post comparison, community-engaged hybrid type 2 effectiveness-implementation study design will focus on the implementation of Navicare at three new sites as well as monitoring implementation at an existing site established since 2021. The rationale for choosing a type 2 design aligns with criteria reported for these designs [18]. This includes having some but not strong evidence of positive effects of components of the Navicare model on mental health, the likely need for adaptation of the intervention across sites, some existing evidence regarding barriers and facilitators to implementation, and momentum to evaluate Navicare to respond to urgent community needs whilst being implemented. A stepped wedge design with staggered multiple baseline implementation combines repeated measurement and analysis of outcomes within community sites with those captured over time across all sites [18] (Fig. 2). This design is well-suited to pragmatic evaluation of whole-of-community interventions where implementation is likely to do more good than harm, and withholding the intervention via randomisation would be seen as ethically, socially or practically unacceptable [19]. We considered this design to be ideal for evaluating the implementation and effect of Navicare across interconnected communities and where uptake of service access may increase over time.

A community-engaged implementation approach includes community-academic partnerships and community-based participatory research methods [20, 21]. Adopting such an approach was seen as a critical component of implementation success and sustainability by participants in our pilot work in the Region [9]. This community-engaged approach adopts the principles of iterative engagement of diverse stakeholders through all study phases, partnering in implementation decision making and planning, valuing community strengths, tailoring to local context, evaluating meaningful outcomes, and using pragmatic flexible implementation approaches. Besides improving study execution (including implementation, participant recruitment, and data collection), participatory methods can also improve mental health outcomes, collaboration between diverse stakeholders and researchers, and lead to system change [22].

Implementation outcomes and potential mechanisms that influence the study outcomes (including community readiness) will be the focus of this evaluation. A pre-post effectiveness study will use routinely collected data gathered before, during and after Navicare implementation to determine effectiveness of the Navicare model on health service and client outcomes in the region at a population level. Additionally, a longitudinal contextual assessment across the study period will dynamically map the contextual factors influencing Navicare delivery, the need for adaptation and sustainability. It is expected that this evaluation will lead to further refinement or suggestions for refining Navicare including recommendations for implementation strategies that pertain to other rural and remote sites in scaling up the model beyond the study.

### Theoretical approach

Five implementation science frameworks underpin the study.

#### Exploration, preparation, implementation and sustainment (EPIS) framework

EPIS was selected as it was developed based on literature about the implementation of innovations in public sector social and allied health service systems including mental health in the United States [23]. The framework has been applied extensively in mental health sectors, including in Australia [23], as well as in cancer control in sub-Saharan Africa [24]. It also has a considerable focus on factors in the outer context (e.g., health system), and bridging factors that interconnect the inner and outer context, both of which were identified as critically important determinants in our initial research to develop the intervention with community members [9]. The influence of other EPIS domains, the inner context and the innovation being implemented [16] will also be examined. EPIS is particularly well suited to examining the role of implementation power, equity and person-oriented recovery [8]—often neglected factors in evaluating the success of mental health interventions [25].

In recognition of the importance of the exploration and preparation phases of EPIS in the initial co-design work [9] and to the success of innovations more broadly [26], our study design includes these phases prior to the implementation of Navicare at any new site. As a determinants and process framework, use of EPIS will allow for a prospective understanding of determinants and mechanisms of implementation, and tailoring of implementation strategies to overcome these determinants mapped to the EPIS phases [26], scaling-up across multiple sites, and evaluating interconnections and variance across factors and phases [8]. The economic evaluation, engagement with stakeholders, and adaptation of the implementation to the local context at each site will also be mapped to the EPIS phases to determine the activities, costs and extent of engagement and adaptation in each phase and across phases.

**Fig. 3:**
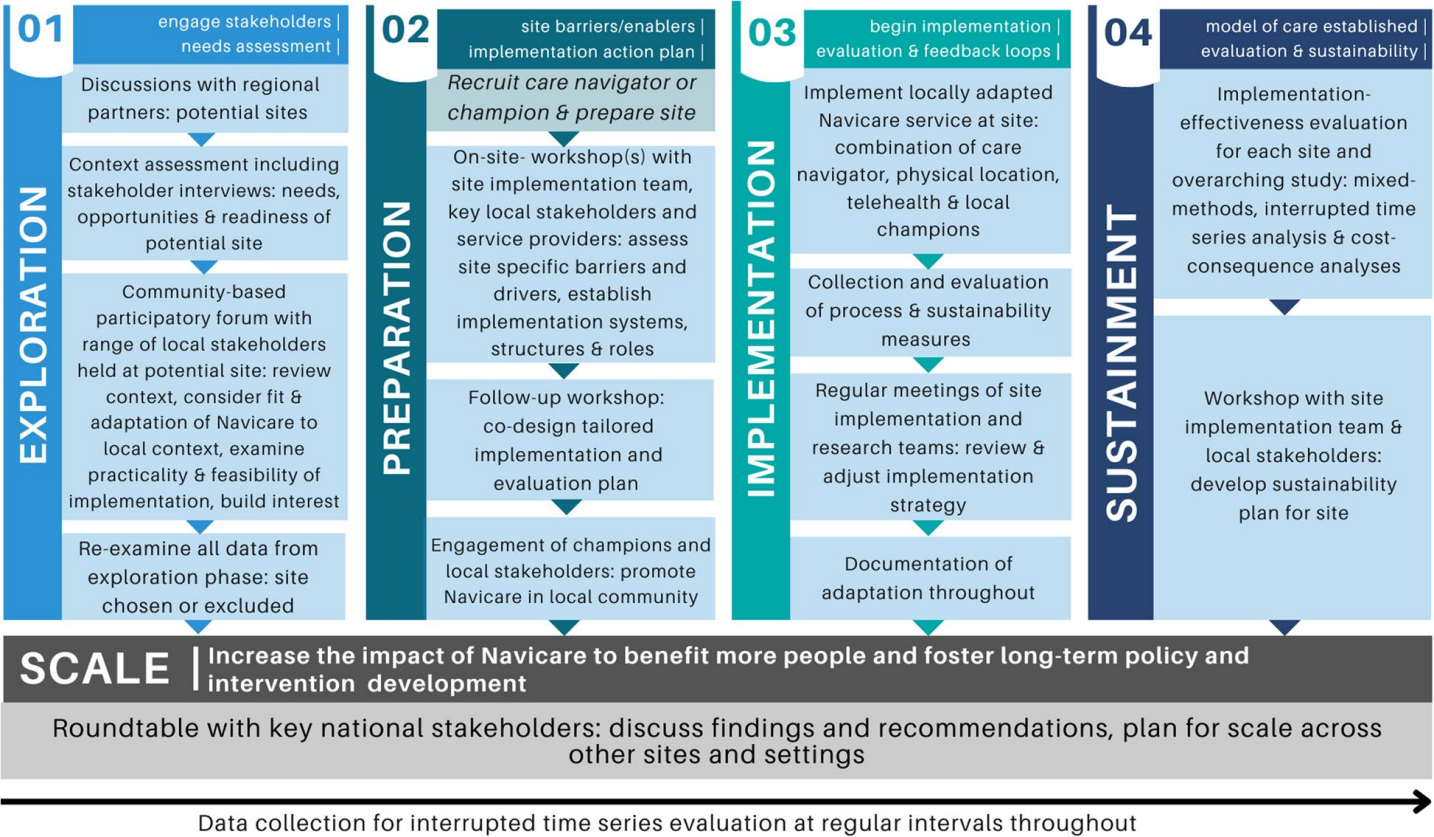
Study activities by EPIS phase for each site and scaling-up

All four EPIS phases and activities will be sequentially applied at the three new study sites. New sites will be identified through consultation with key stakeholders as part of our community-engaged approach in the exploration phase. The proposed timeline for each phase at each site is outlined in Fig. 2, however we expect the duration of time spent in the exploration and preparation phases may change based on circumstances at the local sites. Study activities by EPIS phase for each site are outlined in Fig. 3, including community consultations, workshops, and site implementation stakeholder groups. The exploration and preparation phases have already been completed at the existing site where implementation commenced in 2021. For this existing site, evaluation will be conducted in the implementation and sustainment phases only. Study activities in the exploration, preparation and implementation phases will be the same across all new sites (Figs. 2 and 3). This is consistent with EPIS, in recognising that the effectiveness of implementation is influenced at least in part by activities in the exploration and preparation phases [26]. Implementation strategies, however, may be adapted and tailored for sites based on the localised contextual factors identified in the Exploration and Preparation phases at each site.

#### Implementation Outcomes Framework (IOF)

Proctor’s framework was selected to guide selection of the implementation outcomes for this project and includes feasibility, acceptability, appropriateness, adoption, cost of implementation, fidelity and sustainability [27, 28]. Definitions of each of the constructs along with their operationalisation in the study are outlined in Table 1.

**Table 1:** Implementation outcomes: description, data type, source and timepoint of measurement

Outcome	Description of the outcome	Data type, source and timepoint of measurement
Sustained Access (co-primary and secondary outcome) ^a^	Total number of new eligible persons with or at risk of mental ill-health or their carers who contact Navicare to seek assistance from or through Navicare each month (co-primary outcome). Total number of new eligible persons with or at risk of mental ill-health or their carers who are referred to Navicare, engage with Navicare referral services (i.e., mental health service encounters) or use Navicare telehealth services (secondary outcomes)	Quantitative: Navicare service data Timepoint: Monthly across sites during the implementation and sustainment phases. For the existing site the 12 months prior to the sustainment period will be included. The sustainment period will be compared to the implementation period across sites
Feasibility	The extent to which the Navicare service can be successfully used or carried out at new sites. ^a^	Qualitative: Service user, provider and community member interviews; Care Navigator implementation diaries; field notes, relevant documents, and observation Timepoint: Up to monthly across the implementation (12-months) and sustainment (12-months) phases, with data collected during each EPIS phase
Acceptability	The acceptability of the Navicare service and evaluation by service users, caregivers and providers including satisfaction with the care navigation component and referred services including cross-cultural acceptability and safety. ^a^	Quantitative: NAVSAT completed by service users or caregiver Qualitative: Interviews and/or focus groups with service users, caregivers and providers; field notes and documents Timepoint: Up to 6-months after Navicare intake or 3-months after last contact with Navicare for service user data including NAVSAT and interviews
Appropriateness	The extent to which the Navicare service is perceived as suitable, compatible, useful and practical and aligns with the values, needs and experiences of the target population and context. ^a, b^	Qualitative: Interviews and/or focus groups with service users, providers and community members; Care Navigator implementation diaries; and field notes, relevant documents and observation Timepoint: As per feasibility
Adoption	Service level assessment of actions to engage with or use the Navicare service ^a^, including frequency, timing and quality of communication and referrals, occasions of service (number, timing), % available services accessed by service user (e.g., counselling), and extent of service provision	Quantitative: Navicare service and project data Qualitative: Care Navigator implementation diaries, project records and meeting minutes Timepoints: As per feasibility
Fidelity	The extent to which the Navicare service is delivered and received as intended. This includes the core components of the intervention (fidelity). ^a^	Quantitative: A fidelity-adaptation tool Qualitative: Care Navigator implementation diaries, workshops, focus groups; and interviews with care navigators, providers and community members Timepoints: As per feasibility
Sustainability	The extent to which the Navicare intervention (or a modification of the intervention) was continued or planned to be continued at the end of the study, and barriers and facilitators of sustained use. ^a^	Quantitative: PSAT completed by Care Navigators and research staff Qualitative: Interviews with consumers and service users, field notes, project records and relevant documents Timepoints: Throughout the study period with PSAT completed at the end of one year of implementation at each site
Cost of implementation	The cost of implementing the Navicare service at each new site, including service costs, costs related to the implementation strategies used, and the location of the Navicare service. ^a^ The costs relating to both implementation strategies and intervention delivery will include salary, facilities, hiring, staff education and training, equipment, materials and resources	Quantitative: Navicare service data, project records, fidelity-adaptation tool, data obtained from health services databases (QHEDC & QHAPDC) Qualitative: Care Navigator implementation diaries, interviews related to cost and study activities Timepoint: Throughout the study period
Scaling-up	The suitability of Navicare as an evidence-based intervention designed for regional Queensland, for implementation in other similar settings	Qualitative: Roundtable and all other relevant qualitative study data Timepoint: During final year of the study
Implementation	Implementation processes and contextual factors (e.g., duration, quality, barriers and enablers and other contextual factors)	Quantitative: Service and project records, sociodemographic data, fidelity-adaptation tool Qualitative: Care Navigator implementation diaries, stakeholder interviews, field notes based on observation, discussion with service providers and users Timepoint: Throughout the study period

*Abbreviations*: *EPIS* Exploration, Preparation, Implementation, Sustainment framework, *NAVSAT* Navigation Satisfaction Tool, *PSAT* Program Sustainability Assessment Tool, *QHEDC* Queensland Hospital Emergency Department Data Collection, *QHAPDC* Queensland Hospital Admitted Patient Data Collection

^a^Based on Proctor’s Outcomes and Indicators [27, 28]

^b^Based on Rogers’ definition of appropriateness [29]

#### Framework for Reporting Adaptations and Modifications – Enhanced (FRAME) and Evidence-based Implementation Strategies (FRAME-IS)

FRAME consists of eight aspects including whether the intervention adaptation was planned or unplanned, the extent the adaptation is fidelity consistent and the intent or goal of the modification [30]. The FRAME-IS consists of four core elements to guide documentation: (1) describing the evidence-based practice, implementation strategy and adaptation; (2) what is adapted; (3) the nature of the adaptation; and (4) the rationale for the adaptation [31]. Delivery and adaptation of the Navicare intervention and implementation strategies will be guided by FRAME and FRAME-IS respectively across the study period and sites. This will assist in determining modifications to Navicare and implementation strategies that were associated with successful versus unsuccessful implementation as well as core implementation strategies [30, 31].

#### World Health Organisation ExpandNet framework (ExpandNet/WHO)

The validated ExpandNet/WHO framework consists of the elements of the intervention, user organisation(s), environment, resource team, and scaling-up strategy and is guided by the principles of systems thinking, a focus on sustainability, the need to determine scalability, and respect for gender, equity and human rights principles [32, 33]. The framework will be utilised in developing a scalability strategy with stakeholders during a roundtable workshop.

### Implementation study component methods

This study component will address aims 1 to 4.

#### Population

The inclusion criteria for service user or carer participants (over the age of 16 years) are, being a current or past user of Navicare in any of the site locations or broader mental health services in the region. Service providers and other participants will be included if they are a current or past service provider through Navicare or are connected with Navicare as a relevant stakeholder, including Care Navigators, community members, government agencies and policy makers. Roundtable stakeholder participants will include: experts in mental health across Australia (including those in regional mental health and recovery-based programs); at least two people with lived experience of mental ill-health; a Care Navigator from Navicare; and representatives from non-government and government mental health agencies, including policy makers.

#### Recruitment

Potential individual participants (e.g., service users, carers and service providers) will be contacted by a member of the study team or a study partner with an existing relationship to the provider or user, but not by the service providers of users (e.g., psychologists, social workers). Initial contact will be made in the form of an email, newsletter or flyer outlining the intent of the research and information on how to participate. Potential participants who are interested in taking part will be required to contact the research team if they would like to participate, following which information and consent forms will be sent to them. Potential participants who respond to a request to participate may be asked to complete screening questions as part of the process to ensure diverse representation in qualitative study activities (e.g., age, gender). Participants must provide written consent prior to engaging in community consultations, interviews, implementation diaries, the roundtable, and workshops. Non-English speaking potential participants and people with language impairments will be able to participate if they have a support person who is able to assist with translation and language assistance.

#### Data collection

The sampling approach for qualitative data collection will be convenience sampling initially, followed by purposeful sampling then theoretical sampling [34] with diverse representation sought as part of our recruitment strategy (Table 2). Routinely collected deidentified Navicare service-level data will be accessed, including sociodemographic data and telehealth usage. Sociodemographic data will also be collected from Navicare users and providers who consent to participate in interviews, focus groups and surveys.

**Table 2:** Characteristics of stakeholder groups sought for diverse representation

Service users	Service and support providers
- Ages including young people 16 to 18 years and 18 to 24 years, older persons - Non-binary genders - Type of service user (e.g., local resident, Drive In Drive Out/ Fly In Fly Out worker) - Previous experience with Navicare (those with lived experience and no lived experience) - Socioeconomic circumstances (e.g., employed, not employed) - Diverse cultural and linguistic backgrounds - With and without lived experience of mental ill-health - Service users of Navicare and other services - Community members who have not accessed Navicare services but have lived experience of mental ill-health - Parents and carers of people with mental ill-health	- Representation from local government, Primary Health Network, community support organisations like local sporting organisations, telehealth providers, general practitioners and allied health professionals, educators, policy makers, Lifeline, alcohol and other drugs support workers, community housing - Local businesses (mining and industry) - Diverse cultural and linguistic backgrounds

#### Co-primary outcome

Our novel co-primary outcome measure of sustained access to Navicare was identified in our initial intervention co-design work with communities as being of great importance [9]. This outcome will be evaluated using the total number of new eligible persons with or at risk of mental ill-health or their carers who seek assistance from or through Navicare each month either directly or by provider referral. These data will be obtained from routinely collected Navicare service data. Access will be measured monthly across the study, comparing implementation (12-months) and sustainment (12-months) phases across all new sites, with commencement defined according to when the first client is seen physically at each new local site (see Fig. 2). We consider this outcome to bear similarity to Proctor’s outcome of equitable access, with access hypothesised to combine the outcomes of penetration, sustainability and fidelity [28].

#### Implementation outcomes and adaptation

Mixed methods data will contribute to the outcomes of acceptability, adoption, fidelity, sustainability, cost of implementation, implementation, and adaptation. Qualitative data will contribute to feasibility, appropriateness and scaling-up. The following outcome measures and approaches will be used, with measurement timepoints and further details outlined in Tables 1 and 3. Outcome measures to be used include the *Navigation Satisfaction Tool (NAVSAT)* [35], *Program Sustainability Assessment Tool (PSAT)* [36] and a *fidelity-adaptation tool* to be developed as part of the study. The tool will document core non-adaptable and modifiable intervention components and the extent to which Navicare intervention activities are delivered as planned or adapted to meet local community needs, and explore reasons underpinning adaptations (detailed in Table 3).

**Table 3:** Protocol outcome measures

Outcome measure	Outcome	Time of data collection and people completing	Description, scaling and content	Psychometric and other properties
Navigation Satisfaction Tool [34]	Acceptability in terms of satisfaction with care navigation and referral services used by service users	Navicare service users will complete up to 6-months after intake to Navicare or 3-months after last contact with Navicare	22-items rated on a likert scale from 1 (extremely dissatisfied, extremely unhelpful, or extremely small degree) to 7 (extremely satisfied, extremely helpful, or extremely large degree). Topics covered include the navigator’s ability to listen and understand service user concerns, the mental health system, information about potential treatment options, intake procedures, navigator’s helpfulness, likelihood of recommending the service, and overall satisfaction with the service, as well as levels of perceived effectiveness and satisfaction with aspects of the referred service [34]	Acceptable reliability, face validity, and predictive validity for service users of care navigation services [34]
Program Sustainability Assessment Tool [35]	Sustainability at the program level	Navigators and the research implementation team will complete at the end of one year of implementation at each site	40-items rated on a Likert scale from 1 (‘to little or no extent’) to 7 (‘to a very great extent’). Eight domains are environmental support, financial stability, partnerships, strategic planning, communications, program adaptation, program evaluation, and organisational capacity	Good fit of the data with the 8 domains using confirmatory factor analysis and acceptable internal consistency when used in chronic disease programs [35]
Study specific fidelity-adaptation tool	Fidelity and adaptation	Research implementation team will complete throughout the study. Member checking by the original co-developers of the Navicare model and implementation strategies	Items specific to the study will be developed based on FRAME, FRAME-IS, elements of adaptation [36], and the proportion and duration of activities in each phase similar to the Stages of Implementation Completion measure [37]. Data from multiple data sources will be mapped to the tool items (e.g., from semi-structured interviews, community consultations, workshops, observations at forums or site visits, implementation diaries and documents referred to in data collection activities). Member checking will be conducted against originally developed core intervention components and implementation strategies [36, 38]	Not applicable

*Abbreviations*: *FRAME* Framework for Reporting Adaptations and Modifications – Enhanced (FRAME), *FRAME-IS* Framework for Reporting Adaptations and Modifications – Enhanced Evidence-based Implementation Strategies (FRAME-IS)

*Implementation diaries* will be used to collect information on everyday work of Navicare to report on the process of care navigation as well as being a tool to capture resource use, and barriers and facilitators to implementation. Implementation diaries will be completed by Care Navigators throughout the implementation and sustainment phases to allow an in-depth understanding of project implementation by capturing project evolution and real-time insights while reducing self-reporting bias [39, 40]. The structure of the diaries will be based on the EPIS framework constructs as well as implementation outcomes relevant to the study. Implementation diaries will be completed monthly in the implementation and sustainment period at the new study sites, hosted in Redcap, an electronic data capture tool [41].

*Context assessment and evaluation of contextual factors* (for example, barriers and facilitators, mechanisms) will be conducted using a hybrid inductive-deductive approach. Constructivist methodology [42, 43] will underpin the inductive approach, and EPIS study phases will inform the deductive approach to capture how these factors influence the findings. Using constructivist methodology has been reported to promote a multi-layered approach and can assist in building a holistic understanding of context as dynamic across the study rather than static, capturing interactions within context and maximising uptake and sustainability when used to inform subsequent phases of implementation [42]. Context and interactions between context, intervention, and implementation will be used to prospectively inform subsequent study phases and will be analysed separately from implementation processes [42]. Data sources will include semi-structured interviews, community consultations, and workshops with service users, carers, and service providers at all sites; and observations at forums or site visits. Field notes and memos will be made in relation to all data sources.

*Scaling up* nationally will be prepared for using a national roundtable forum in the final year of the study, with subsequent email or virtual feedback from participants to summarise themes, recommendations and evidence-based practice gaps for sustainability and national scaling of Navicare [32]. ExpandNet resources will be used including the ExpandNet/WHO framework for scaling up [32, 44]. A scalability strategy will be co-developed with stakeholders using the ExpandNet/WHO Nine Steps for Developing a Scalability Strategy [32] if evidence collected from the four study sites supports success aligned with the study aims. The actions and findings throughout the preceding four EPIS phases will directly support this process and provide evidence of the scalability of Navicare nationally.

#### Data analysis

The co-primary implementation outcome of sustained access will be analysed quantitively using a regression model. A hybrid mixed methods approach integrating inductive and deductive qualitative analyses will be used for qualitative data. Inductive analyses will be informed by constructivist grounded theory [45] and deductive analyses will be conducted using framework analysis. These analyses will involve constant comparison (systematic comparison across participants) throughout the study and developing and charting data into a working framework [46]. The working framework will be based on the EPIS, study sites, and ExpandNet/WHO frameworks, the fidelity-adaptation tool [47], or other factors identified from the inductive analysis. Themes will be summarised to generate recommendations for scaling up Navicare or alternate mental health access and support programs, and to highlight evidence-based practice gaps.

Quantitative data analysed descriptively or using regression models will be combined with qualitative data to report on the implementation outcomes, context and contextual factors influencing implementation, and outcomes related to the process of applying EPIS (for example, variability in the timing of the exploration and preparation phases across sites prior to new site implementation) where relevant. As part of this mixed methods approach, qualitative and quantitative data will be used for sampling, triangulation, elaboration, expansion and development of the data [48]. The latter will involve using the findings to inform the development of implementation strategies. The process of combining the qualitative and quantitative data is expected to involve merging, connecting and embedding both types of data [49]. Trustworthiness of qualitative and mixed methods analyses will be maintained using field notes, reflexive journaling, memoing, and member checking of data [43, 45, 49]. Visualisation techniques (for example, causal loop diagrams and social network analysis) will be used to report on and provide feedback during the study on the study outcomes (for example, service access) and mechanisms of action of the implementation [50–52].

Cost of implementation will be evaluated across the study sites as well as for each EPIS phase and will include engagement of a local working group during preparation and implementation. Regression models will be used to explore the association between quantitative outcomes (for example, fidelity using the fidelity-adaptation tool with the implementation outcome of sustained access).

#### Implementation strategies

It is expected that multiple implementation strategies will be selected, implemented, and tailored to each site. These strategies are likely to include community and stakeholder engagement and access to local infrastructure. The *Expert Recommendations for Implementing Change (ERIC)* will be used to describe strategies, with FRAME-IS used to guide documentation on how strategies were adapted in the broader context of rural and remote Australia [31, 53]. Other potential strategies not captured by ERIC will be identified and described using the hybrid inductive and deductive data analysis approach (including the use of EPIS to identify barriers and facilitators).

### Effectiveness study component methods

This study component will address aim 5.

#### Population

People with mental health related hospital data from public hospitals in the Greater Whitsunday Region and major regional hospitals situated just outside the Region will be included.

#### Data collection

Local and district hospital level data from the Queensland Hospital Admitted Patient Data Collection (QHAPDC) and Queensland Hospital Emergency Department Data Collection (QHEDC) will be obtained for new sites for the 24-months prior to commencement, during the implementation period of 12-months, and for 12-months in the post-implementation (sustainment) period. The longer length of pre-implementation data will be used to account for seasonal patterns in this control period. For the existing site, QHAPDC and QHEDC data will be collected for 12-months pre-implementation to avoid, as much as possible, the major disruptions due to the COVID-19 pandemic in the first half of 2020, and for the implementation period will be collected from October 2021 when the first client accessed the service. Post-implementation data will be collected from July 2025 at the existing site.

#### Co-primary and secondary outcomes

Outcomes will include time in an emergency department for mental ill-health (co-primary outcome), and mental health crisis-related Emergency Department attendances and admitted overnight hospitalisations for mental ill-health (secondary outcomes).

#### Data analysis

A pre-post comparison analysis will be used to examine if Navicare has a greater effect than any underlying secular trend. This will be achieved by fitting a generalised linear mixed model, to model the monthly rates of time in the emergency department (using a Gamma or normal distribution depending on the data), mental health emergency department presentations (using a Poisson distribution), and admitted overnight hospitalisations (using a Poisson distribution) [54]. Random intercepts will account for the non-independence of data from the same facilities. For sustained service access (co-primary outcome) and adoption, the trend before and after formal support for Navicare ends will be modelled (implementation to sustainment periods, Hypothesis 1). Residuals of the models used will be checked to assess the model’s validity, with histograms to check for bimodality and outliers, and with autocorrelation over time assessed with the Durbin Watson test, and adjustments for underlying trends and seasonality.

### Economic evaluation methods

This study component will address aim 6.

#### Population

The economic evaluation will synthesise relevant service and population level resource use data.

#### Data collection

Program and associated implementation related resource use will be estimated using service and project records, including implementation diaries. Staff time will be costed using estimates of salary ranges, while the cost of materials, equipment, consumables and travel will be valued using market prices. Potential cost savings from reduced emergency department presentations and overnight hospitalisations in the post-implementation period will be estimated using an approach that is consistent with the effectiveness analysis.

#### Data analysis

A* cost-consequence analysis* framework will be used to estimate the health service resource use and costs [55] associated with delivery of Navicare and associated implementation strategies [56] during the study period. Results will be descriptively summarised across disaggregated resource use and cost categories and presented alongside associated measures of program effectiveness to produce estimates of the ‘cost per service user’ and ‘cost per occasion of service’ to inform future planning and decision making. Uncertainty will be represented as 95% confidence intervals using non-parametric bootstrapping.

### Sample size estimates

Sample size for the co-primary outcome of sustained access has been pragmatically based on the monthly numbers of new people in contact with Navicare for mental health or wellbeing services in the implementation and sustainment phases. Pilot work at the existing study site conservatively indicates that approximately 17 new adults or carers with mental ill-health (or at risk of mental ill-health) will contact Navicare monthly during the implementation phase at the existing site and a new site of similar size. Extrapolating this level of access to two expected smaller sites, approximately 13 new adults or carers with mental ill-health are expected to contact Navicare monthly during the implementation period at each of these sites. Sustainment phase contact numbers are unknown in advance but we expect the number of new people with mental ill-health (or at risk of mental ill-health) contacting Navicare to increase or at least stay the same in the sustainment period compared to the implementation period across the sites. Based on these estimates we have 94% power to detect a 20% increase in new Navicare client contact numbers in the sustainment period compared to the implementation period using a one-sided test.

The number of service consumers and service provider participants in qualitative data collection involving community consultations, workshops, and interviews is difficult to determine in advance of the analysis which will be ongoing throughout the project. However, a minimum number of 20 service providers and 30 consumers across the study has been pragmatically determined for planning purposes and to ensure diverse stakeholder representation (see Table 2).

### Sociodemographic and health services data and feedback of study findings

Sociodemographic data will be used to describe samples and may be used to control for factors in quantitative analyses and as sensitising concepts in qualitative analyses. Sociodemographic data about individual study participants will be collected for study purposes. Routinely collected aggregated sociodemographic service data from Navicare will also be collected (for example, engagement with telehealth services at the site level) and will be used to describe the services accessed by study participants. QHAPD and QHEDC databases will be used to describe hospital services accessed for mental health in the region. The extent of feedback of study findings to participants, service providers, community members and key stakeholders based on the study findings will be recorded throughout the study.

## Discussion

This multisite, theory-informed, mixed methods evaluation will result in a context assessment across study sites, evidence of effectiveness of the intervention and implementation strategies at a population level, an economic evaluation, and national scaling strategy if indicated. A process evaluation of community-engaged implementation, tailored to individuals and local communities in rural and remote Australia using EPIS will be completed, with specific outcomes in three Queensland communities. Targeting all people in communities who are at risk of or with mental ill-health as part of the intervention and implementation is unique, and of importance, as the project should result in a greater understanding of the strategies that foster service networks and promote access; traversing ages, diagnostic groups and service levels (for example, primary, community, and hospital healthcare). This differs to most other mental health programs that have targeted specific populations, for example, the Mates in Mining program [57]. Our community-engaged approach, partnering with diverse stakeholders at all levels and phases of implementation, with a particular focus on exploration and preparation, is also unique in this context. Not only has such an approach been highlighted as important by local communities in pilot work, but it can also help equalise power imbalances, support trust building, mutually benefit community and research partners, and better incorporate practical community knowledge and priorities into the implementation process [22, 58]. Evidence also suggests such an approach can have positive effects on program sustainability, community empowerment and health outcomes [22, 59].

This study will provide some of the first evidence of the effectiveness of implementing a mental health care navigation model that includes tailored implementation strategies in rural and remote Australia. Evidence from systematic reviews supports the effectiveness of components of the model of care including telehealth and local champions [60, 61]. Recent evidence and policy recommendations also support the approach to mental health care in the current study being informed by local context and tailored to individual and local needs [62–64], as rural and remote communities are not homogeneous [5]. These policy recommendations have arisen from the Orange Declaration of Rural and Remote Mental Health 2019 in Australia [65]; as well as evidence of health system building blocks in rural and remote health internationally, aligned with the World Health Organization Framework for Action on Strengthening Health Systems to Improve Health Outcomes [66, 67].

A potential limitation is the lack of a control group for the evaluation. However, obtaining an appropriate control group for this type of intervention which has already been implemented is challenging. Targeting rural and remote areas in a state in Australia may also limit generalisability of the findings to metropolitan and rural and remote areas in other states of Australia and internationally. Further, using routinely collected data for health service outcomes is also a potential limitation in that the quality of the data is unknown, however this is also a strength in that the potential burden to people with mental ill-health can be minimised and disadvantages related to measurement in a research context alone may be addressed [68]. Population level effects can also be determined. Finally, we have incorporated a measure of satisfaction with care navigation, but recognise that satisfaction may not be an important measure to evaluate client treatment outcomes [69]. The relevance of satisfaction-based measures would be strengthened by further research that examines correlations with clinical outcomes in the study context.

## Conclusion

The dramatic decline in mental health worldwide in the early COVID-19 pandemic years has not recovered, with the poorest mental health in wealthier Anglosphere countries like Australia, highlighting this international and national health priority. For people with mental ill-health in rural and remote Australia, difficulty accessing appropriate and timely mental health services is a pressing concern. This study will evaluate a novel co-primary implementation outcome of sustained access to a mental health care navigation service, for people in four rural and remote communities in Australia. More broadly the study will evaluate the implementation, effectiveness and cost-consequences of the care navigation service (Navicare). The potential for scaling up Navicare nationally will be evaluated which may inform a national strategy if indicated. Findings from the research will inform recommendations for using EPIS as a framework that can be applied dynamically over time to prepare for and tailor implementation over the course of a study, potentially reducing the complexity of using multiple theories and frameworks.

## Data Availability

The dataset generated and analysed in the study may be available if appropriate permissions are obtained (by those seeking to access the data) from the first author and Townsville Health Human Research Ethics Committee.

## Abbreviations

EPIS: Exploration, Preparation, Implementation and Sustainment framework
FRAME-IS: Framework for Reporting Adaptations and Modifications to Evidence-based Implementation Strategies
NAVSAT: Navigation Satisfaction Tool
PSAT: Program Sustainability Assessment Tool
QHAPDC: Queensland Hospital Admitted Patient Data Collection
QHEDC: Queensland Hospital Emergency Department Data Collection
